# Collagen-hyaluronic acid hydrogel with embedded chondrocytes as a platform for modeling early stages of endochondral ossification *in vitro*

**DOI:** 10.1177/09636897251409464

**Published:** 2026-02-13

**Authors:** Marina Malić, Martina Doubková, Šimon Pražák, Antonín Brož, Kristýna Havlíčková, Věra Jenčová, Daniel Hadraba, Lucie Bačáková

**Affiliations:** 1 Laboratory of Biomaterials and Tissue Engineering, Institute of Physiology of the Czech Academy of Sciences, Prague, Czech Republic; 2 Nanotechnology Centre, CEET, VSB—Technical University of Ostrava, Ostrava, Czech Republic; 3 First Faculty of Medicine, Charles University, Prague, Czech Republic; 4 Second Faculty of Medicine, Charles University, Prague, Czech Republic; 5 Faculty of Science, Humanities and Education, Department of Chemistry, Technical University of Liberec, Liberec, Czech Republic; 6 Laboratory of Advanced Microscopy and Data Analyses, Institute of Physiology of the Czech Academy of Sciences, Prague, Czech Republic

**Keywords:** *in vitro* model, hydrogel, collagen, endochondral ossification, hMSCs, hyaluronic acid

## Abstract

Tissue engineering strategies aim to create bone models to enhance our understanding of bone development and improve bone repair. Endochondral ossification (EO), where cartilage is replaced by bone, is difficult to replicate. We developed a collagen–hyaluronic acid (HA) hydrogel system for a three-dimensional (3D) co-cultivation of human chondrocytes, human mesenchymal stem cell (hMSCs), and human umbilical vein endothelial cells (HUVECs) to model the early stages of EO. To our knowledge, this specific tri-culture configuration within such a hydrogel composition has not been previously reported, providing a novel platform for studying EO-related cellular and matrix interactions *in vitro.* Differentiation and migration of hMSCs and HUVECs were studied in collagen I (Col I) hydrogels, with or without the addition of collagen II (Col II), HA, and chondrocytes. Col II and HA enhanced cell osteogenic differentiation, as documented by an increased *COL1A1* and *ALPL* gene expression and enhanced alkaline phosphatase activity. Chondrocytes further promoted osteogenic cell differentiation and temporarily enhanced hypertrophic and angiogenic signaling as evidenced by elevated *MMP13* and *VEGFA* expression. Furthermore, elevated *SOX9* expression and Alcian blue staining indicated a chondrogenic-supportive environment. These findings highlight the synergistic effects of osteogenic and chondrogenic signals, emphasizing the potential of combining Col I and Col II, HA, and essential cell types in hydrogels to optimize bone and cartilage tissue engineering.

## Graphical abstract

**Graphical Abstract:**
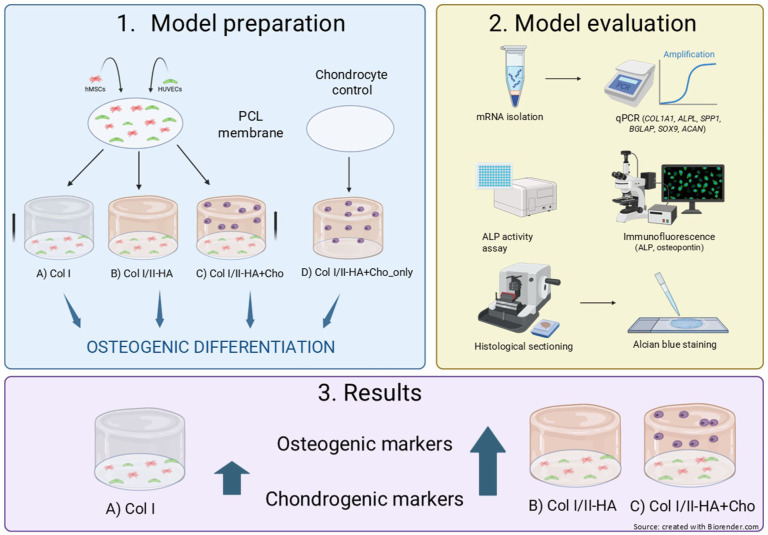

## Introduction

Three-dimensional (3D) *in vitro* models have become essential tools for studying biological processes in a physiologically relevant environment. These models provide a more accurate representation of tissue architecture, cell-cell interactions, and extracellular matrix (ECM) composition compared to traditional two-dimensional (2D) cultures^1^. Developing bone-like *in vitro* models is crucial for understanding early bone development, post-injury ossification, and improving bone repair and grafting strategies^2^. While several *in vitro* systems have been designed to model bone metabolism and vascularization (see Ehnert et al.^3^ for a review) or bone remodeling (see Owen and Reilly^4^ for a review), replicating the *in vivo* process of endochondral ossification (EO) remains a significant challenge.

EO is one of the two main mechanisms of bone formation and repair^5^. It begins with mesenchymal condensation, where mesenchymal stem cells (MSCs) aggregate and differentiate into chondrocytes, which proliferate and form a hyaline cartilage template. Within this structure, chondrocytes organize into zones: low-proliferative chondrocytes at the distal ends of condensation that maintain the chondrogenic phenotype and high-proliferative chondrocytes lying in columns toward the center that then undergo maturation. During maturation, central chondrocytes undergo hypertrophy, enlarging up to twentyfold and producing a mineralized matrix that serves as a scaffold for subsequent bone deposition. Blood vessels from the periosteum, along with osteoblasts and osteoclasts, then invade the calcified matrix produced by hypertrophic chondrocytes, replacing it with bone to establish the primary ossification center. The newly formed matrix is subsequently remodeled into cortical bone and a bone marrow cavity, which supports hematopoiesis^6^. While this transformation occurs in the center, the cartilage at the ends of the bone continues to grow and proliferate, contributing to bone elongation. During postnatal development, secondary ossification centers emerge at the distal ends of long bones, completing skeletal development^7^.

Hyaline cartilage, the precursor tissue in EO, consists predominantly of ECM with only 1–2% chondrocytes^8^. Its ECM is rich in water, type II collagen (Col II), and proteoglycans such as aggrecan, which binds to long hyaluronic acid (HA) chains to provide compressive resistance and mechanical stability of cartilage^9–11^. HA is also known to interact with cells and influence cell adhesion, proliferation, differentiation, and ECM turnover^11^. Col II provides tensile strength and structural integrity of hyaline cartilage by forming a fibrous framework that supports the highly hydrated cartilage environment. Of the 28 identified types of collagen, Col II is the main component of cartilage, while type I collagen (Col I) is more prevalent in bones and tendons^12^. The unique composition of hyaline cartilage, characterized by a high water content, collagen, and proteoglycan networks, yields a hydrated, gel-like structure that closely resembles the structure of hydrogels^13^.

Hydrogels are 3D polymer networks capable of retaining large amounts of water. This gives them the ability to mimic the native ECM, making them a widely studied subject in tissue engineering^11–13^. Hydrogels derived from natural macromolecules such as alginate, chitosan, HA, and collagen offer excellent biosafety and biodegradability for biomedical use^14^. Collagen-based hydrogels, in particular, provide a biologically active matrix that promotes the adhesion, migration, and osteogenic differentiation of human mesenchymal stem cells (hMSCs)^12^. Additionally, hydrogels combining collagen and HA have been developed for various biomedical applications, such as drug delivery, cell culture, tissue regeneration, and wound healing, showing enhanced performance compared to using either component alone^11^.

However, developing a 3D *in vitro* model that accurately replicates the complex, multistage process of EO remains difficult. It requires reproducing the dynamic interplay between chondrocytes, osteoblasts, and endothelial cells, along with the gradual transition from a cartilage-like matrix to an osteoid tissue^15^. Although several studies have partially mimicked EO using hMSCs alone^16,17^, or co-cultures with HUVECs^18^ or chondrocytes^19^, these models do not fully recapitulate the cellular and ECM complexity of the native process. Furthermore, while hydrogels resembling the cartilaginous ECM have been extensively explored for cartilage repair^20–23^, their potential in bone tissue formation through EO mechanisms remains largely underexplored. To address these challenges, this study developed models based on collagen–HA blend hydrogels designed to mimic the composition of native hyaline cartilage, allowing us to investigate the osteogenic activity of cells within them. Although developmental processes (e.g., mesenchymal condensation, chondrogenesis, hypertrophy, vascular invasion, and subsequent bone formation) represent critical stages in native EO^6^ and form the basis of many *in vitro* EO models^16–18,24^, our approach was different. Rather than replicating each developmental phase, this study examined how cartilage-associated components (Col II, HA, and chondrocytes) influence osteogenic processes when integrated into a 3D hydrogel system. By doing so, we aimed to explore whether incorporating features of the cartilage microenvironment into a bone-oriented culture system could influence osteogenesis and provide new insights into the osteogenic potential of these biomimetic hydrogels. Our model successfully integrated hMSCs, endothelial cells (HUVECs), and chondrocytes—the three cell types crucial to the native process of EO. This model may offer a closer approximation of the *in vivo* environment, potentially providing valuable insights into the interactions that influence osteogenic differentiation.

Our model was adapted from the skin tissue construct developed by Bacakova et al.^25^, in which a collagen hydrogel was cast onto a nanofibrous membrane and suspended within a cultivation well insert to facilitate nutrient exchange from below. Building on this concept, we used a poly-ε-caprolactone (PCL) nanofibrous membrane as a mechanically stable and permeable support for cell culture. PCL is a biocompatible and biodegradable polyester widely used in tissue engineering, though its hydrophobic surface limits direct cell interactions^26^. To enhance cell attachment, the membrane was coated with fibrin, providing bioactive sites for adhesion^27^. The PCL layer was then seeded with hMSCs and HUVECs. Subsequently, a hydrogel composed of a 3:1 blend of Col I and Col II was cast on top of the membrane to mimic the ECM of hyaline cartilage. As demonstrated in a study by Vázquez-Portalatín et al.^28^, this collagen ratio exhibits superior mechanical properties compared to other blends. Although Col II is the primary component of hyaline cartilage, Col I is more readily available and has already been included in Food and Drug Administration-approved tissue engineering constructs^22^. To more closely mimic the composition of native cartilage, the hydrogel was enriched with HA and embedded chondrocytes, the cells responsible for cartilage production^29^. This cartilage-like hydrogel provided a suitable 3D microenvironment that allowed hMSCs and HUVECs to migrate from the PCL membrane into the hydrogel, mimicking the natural process of EO.

To our knowledge, this specific tri-culture configuration within such a hydrogel composition has not been previously reported, providing a novel platform for studying EO-related cellular and matrix interactions *in vitro*. We hypothesized that incorporating Col II and HA into the hydrogel would enhance chondrogenic and osteogenic activity compared to Col I alone (Col I hydrogel). Furthermore, we expected chondrocytes in the Col I/II-HA+Cho hydrogel to deposit ECM, enhancing the model’s resemblance to native hyaline cartilage and facilitating EO.

## Materials and methods

### Preparation and assessment of PCL membranes

A polycaprolactone nanofibrous material (PCL80, Mw = 80,000 g·mol^−1^) was prepared using the direct current (DC) needle-less electrospinning technique on a Nanospider^TM^machine NS 1WS500U (Elmarco, Liberec, Czech Republic). The polymer solution contained a mixture of PCL80 in a chloroform/ethanol solvent system (ratio 8:2), and electrospinning was performed according to the following experimental details: the spinning electrode was positively charged using a high-voltage DC source (40 kV); the collector was negatively charged (−10 kV); the distance between the spinning electrode and the collector was set at 190 mm; the polymer solution was fed onto the stationary wire spinning electrode using a moving carriage module (metal insert orifice of 0.7 mm) filled with the polymer solution; and the withdrawal speed was adjusted (10 mm·min^−1^) to achieve consistent final areal weights for the various nanofibrous layers. The DC electrospinning device was complemented by an air conditioning unit that ensured stable temperature and air humidity values in the spinning space. The temperature was set at 22 ± 2°C, and the relative humidity was set at 40 ± 2%. The supporting material consisted of 20 g/m^2^ spun-bonded Pegatex S (PFNonwovens, Znojmo, Czech Republic).

**Figure 1.**
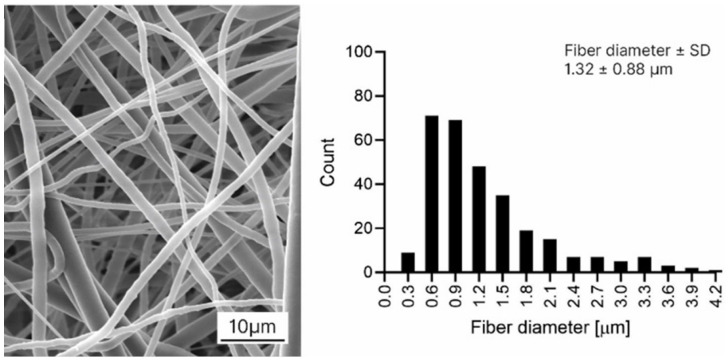
Representative scanning electron microscope micrograph of polycaprolactone nanofibrous material and the corresponding histogram of fiber diameter distribution; scale bar 10 μm.

The morphology of prepared nanofibrous materials was analyzed from pictures obtained with a scanning electron microscope (SEM) Vega S3B EasyProbe (Tescan Orsay Holding a.s., Brno, Czech Republic), and the average fiber diameter was counted from 300 measurements using ImageJ software (NIH, Madison, WI, USA). The PCL 80 nanofibrous material had an average fiber diameter of 1.32 ± 0.88 µm (Fig. 1). The average areal weight of the material was 40 g/m^2^.

The bulk porosity (%) of PCL80 nanofibrous layers was determined by comparing the apparent density of the electrospun samples with the density of the solid polymer (ρ_PCL_ = 1.145 g·cm^−3^). Samples were cut to dimensions of 5 × 5 cm. The mass values of the samples (n = 25 per material) were determined by weighing them on an analytical balance. The thickness values of the samples (n = 25 per material) were measured from cross-section images of the nanofibrous layer acquired with an electron microscope. Nanofibrous samples (n = 4 per material) with dimensions of 0.5 × 3 cm were stretched onto a conical holder and locked in place. Cross-sections of the nanofibrous layer were prepared by cooling the layers with liquid nitrogen. The sample density was obtained by the following equation:

$$\rho_{sample}\left[g\cdot {\mathrm{cm}}^{-3}\right]=\frac{m}{V}=\frac{m}{A\cdot t},$$

where *m* = sample mass [g], *A* = sample area [cm^2^], *t* = sample thickness [cm], *V* = sample volume [cm^3^]. The sample porosity (*P*) was obtained by the following equation:

$$P[\%]=\frac{\rho_{sample}}{\rho_{polymer}}\cdot 100,$$

where *ρ_sample_* = sample density [g·cm^3^], *ρ_polymer_* = polymer density [g·cm^3^]^30^. The obtained values are summarized in Table 1.

**Table 1.** Mean thickness, mean mass, density, and porosity of the PCL80 nanofibrous membranes.

Sample	Mean thickness (cm)	Mean mass (g)	Sample density (g·cm^−3^)	Porosity (%)
PCL80	0.0267	0.0948	0.1418	87.6

### Cell cultivation

The cells used to prepare the 3D model were human bone marrow–derived mesenchymal stem cells (hMSCs), human umbilical vein endothelial cells (HUVECs), and human chondrocytes. The hMSCs were purchased from ScienceCell Research Laboratories (Carlsbad, CA, USA) and were cultivated in Minimum Essential Medium α (MEM α; Gibco, Thermo Fisher Scientific, Waltham, MA, USA) supplemented with 15% fetal bovine serum (FBS; Sebak GmbH, Aidenbach, Germany) and 1% antibiotic-antimycotic solution (ABAM; Sigma-Aldrich, St. Louis, MO, USA). The HUVECs were purchased from PromoCell (Heidelberg, Germany) and were cultivated in Endothelial Cell Growth Medium 2 (EGM-2; PromoCell, Heidelberg, Germany) supplemented with an EGM-2 Supplement Pack (PromoCell, Heidelberg, Germany) and 1% ABAM (Sigma-Aldrich, St. Louis, MO, USA). The human chondrocytes were purchased from Innoprot (Derio, Spain) and were cultivated in a Chondrocyte Medium, supplemented with 5% FBS, 1% Chondrocyte Growth Supplement, and 1% Penicillin/Streptomycin Solution. All components were obtained from Innoprot (Derio, Spain). All three cell types were cultured in an incubator at 37°C with a humidified air atmosphere saturated with 5% CO_2_.

### Preparation of the 3D model of EO

#### Fibrin coating of the PCL membranes and seeding of hMSCs and HUVECs co-culture

The PCL membranes were secured in Cell Crown inserts (Scaffdex Ltd., Tampere, Finland) within 24-well culture plates (TPP, Trasadingen, Switzerland) and coated with a thin fibrin layer to enhance cell adhesion and proliferation, as previously described in a study published by our laboratory^27^. Briefly, after sterilization with ethanol and washing, fibrinogen was adsorbed onto the membranes and activated with thrombin to form a stable fibrin coating. To further enhance cell adhesion, the surface was treated with a fibronectin solution and incubated overnight before cell seeding. A co-culture of hMSCs and HUVECs was seeded on the fibrin-coated membranes at a density of 60,000 cells per well with a 2:1 ratio of hMSCs to HUVECs (40,000 hMSCs and 20,000 HUVECs). The cells were cultivated in complete EGM-2 medium. After 3 days of cultivation, a collagen–HA hydrogel with embedded chondrocytes was prepared on top of the cell-seeded membrane, allowing hMSCs and HUVECs to start migrating into the hydrogel (details provided below).

#### Preparation of hydrogels, cell co-cultivation, and differentiation

A Col I hydrogel with the addition of Col II, HA, and embedded chondrocytes was prepared on top of the hMSCs and HUVECs co-culture 3 days after seeding. The Col I extracted from rat tails was purchased from Sigma-Aldrich (Col I; Corning, NY, USA). Lyophilized chicken sternal Col II (Col II; Sigma-Aldrich, St. Louis, MO, USA) was dissolved in 20-mM acetic acid and sterilized via sterile filtration. The concentration of the resulting Col II stock solution was measured using a BCA Pierce assay (Pierce, Rockford, IL, USA), according to the manufacturer’s protocol. HA (1.5–1.8 × 10^6^ Da; Sigma-Aldrich, St. Louis, MO, USA) was dissolved in 1× phosphate buffer saline (PBS) to a concentration of 10 mg/mL.

**Figure 2.**
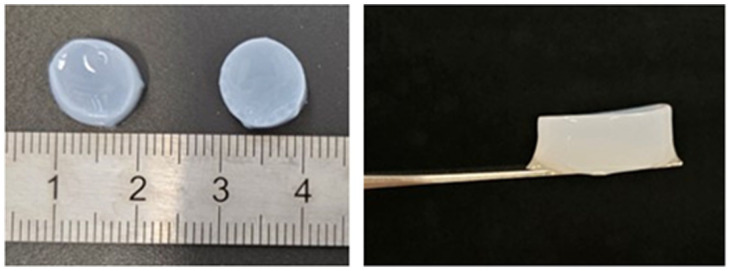
Hydrogels after 1 h of polymerization at 37°C in a humidified atmosphere with 5% CO_2_ in the air.

The final collagen suspension concentration was prepared at 4 mg/mL using a mixture of Col I and Col II at a 3:1 ratio (3 mg/mL Col I to 1 mg/mL Col II). A suspension of HA was added to the mixture to achieve a final concentration of 2.5 mg HA/g hydrogel, consistent with the concentration found in the native articular cartilage (0.5–2.5 mg/g wet tissue)^31^. The pH of the solution was increased to 7.4 by adding 10× PBS and 1 M NaOH, and deionized H₂O was added to achieve the final volume. Chondrocytes dispersed in the chondrocyte medium were added to the hydrogel solution at a density of 500,000 chondrocytes/mL. A total volume of 500 μL of the hydrogel solution was applied to the cell-seeded membrane and left to polymerize for 1 h at 37°C, in a humidified atmosphere with 5% CO_2_. After collagen polymerization (Fig. 2), 1.5 mL of completed EGM-2 medium was added to the samples. After 3 days of cell migration from the fibrin-coated PCL membrane into the hydrogel, the EGM-2 medium was replaced with an osteogenic differentiation medium (DIF), which was a 50:50 mixture of complete MEM α and EGM-2 media, supplemented with 5% FBS (Sebak GmbH, Aidenbach, Germany), M dexamethasone, 50 µg/mL ascorbic acid, 10 mM β-glycerolphosphate, and 1% ABAM (Sigma-Aldrich, St. Louis, MO, USA). The model was then cultured in an incubator at 37°C with a humidified atmosphere saturated with 5% CO_2_. The DIF medium was changed every 2 days.

To evaluate the influence of chondrocytes on osteogenic differentiation, a chondrocyte-free hydrogel (Col I/II-HA) was prepared using the same protocol. To assess the roles of Col II and HA, a control hydrogel consisting only of Col I was included. Additionally, Col I/II-HA+Cho_only hydrogels were prepared on fibrin-coated PCL membranes without a pre-seeded hMSC/HUVEC co-culture, serving as a negative control. The composition of the hydrogels used in the experiments is described in Table 2.

**Table 2.** Composition of hydrogels used in the experiments.

Hydrogel type	Hydrogel components			Cell types used	
	Collagen (mg/mL)		HA (mg/g)	hMSC + HUVEC (40,000 + 20,000) On PCL membrane	Chondrocytes (250,000 in hydrogel)
	Col I	Col II			
Col I	4	0	0	✔	**X**
Col I/II-HA	3	1	2.5	✔	**X**
Col I/II-HA+Cho	3	1	2.5	✔	✔
Col I/II-HA+Cho_only	3	1	2.5	**X**	✔

HA: hyaluronic acid; hMSC: human mesenchymal stem cell; HUVEC: human umbilical vein endothelial cells; PCL: poly-ε-caprolactone.

### Hydrogel characterization

#### Gene expression of osteogenic, chondrogenic, and hypertrophy markers

Osteogenic cell differentiation and chondrogenic activity were assessed at the mRNA level using real-time quantitative PCR (qPCR) to measure the expression of specific genes of interest. mRNA was extracted from the hydrogels after 0, 4, 7, 14, 21, or 28 days of osteogenic differentiation in the DIF medium. Total RNA was extracted from cells in the hydrogel using TRI Reagent (Sigma-Aldrich, St. Louis, MO, USA). The samples were washed in PBS, placed in 2-mL tubes containing ceramic homogenization beads (1.4 mm, Qiagen, Hilden, Germany), and 1 mL of TRIzol. The tubes were then homogenized using a Precellys Evolution homogenizer at a speed of 1000 rpm for 30 s (Bertin Instruments, Montigny-le-Bretonneux, France). RNA isolation was then carried out according to the manufacturer’s protocol. Reverse transcription of 300 ng/μL RNA into cDNA was performed using an Omniscript Qiagen Reverse Transcription Kit (Qiagen, Hilden, Germany) and a Random Primer Mix (New England Biolabs, Ipswich, MA, USA) in a T-Personal Thermocycler (Biometra, Göttingen, Germany). Relative mRNA expression was quantified using 5×HOT FIREPol Probe qPCR Mix Plus (Solis BioDyne, Tartu, Estonia) and TaqMan Gene Expression Assays (Applied Biosystems, Foster City, CA, USA), which were labeled with 6-Carboxyfluorescein (FAM) reporter dye specific to human genes. For osteogenic cell differentiation, the following genes were examined: Col I (*COL1A1* gene, Hs00164004_m1), an early differentiation marker; alkaline phosphatase (*ALPL* gene, Hs01029144_m1), an intermediate marker; osteocalcin (*BGLAP* gene, Hs00165814_m1) and osteopontin (*SPP1* gene, Hs00959010_m1), late differentiation markers. To measure the chondrogenic activity of the cells, we examined the expression of SRY-Box Transcription Factor 9 (*SOX9* gene, Hs00165814_m1), an early chondrogenic marker, and aggrecan (*ACAN* gene, Hs00153936_m1), an intermediate marker. Additionally, we measured the expression of matrix metallopeptidase 13 (*MMP13* gene, Hs00233992_m1), that is, a marker of chondrocyte hypertrophy, as well as the expression of vascular endothelial growth factor A (*VEGFA* gene, Hs00900055_m1), that is, a marker associated with both chondrocyte hypertrophy and angiogenesis. We validated the reference genes using the NormFinder software. Beta-2-microglobulin (*B2M* gene, Hs99999907_m1) was identified as the most stable gene across all experimental conditions and was therefore used as the housekeeping (reference) gene. cDNA amplification was carried out in a 10-μl total reaction volume on the LightCycler 480 System (Roche Diagnostics, Indianapolis, IN, USA) under the following cycling conditions: initial denaturation at 95°C for 10 min, followed by 40 cycles of 95°C for 15 s and 60°C for 1 min. The assay was performed in triplicate, and relative mRNA expression was calculated using the 2^−ΔΔCt^ method. Changes in gene expression were determined using the following equation:

$$\Delta \Delta C_{t}={\left(C_{t}^{target}-C_{t}^{B2M}\right)}_{sample}-{\left(C_{t}^{target}-C_{t}^{B2M}\right)}_{calibrator}$$

Gene expression was normalized to the *B2M* housekeeping gene. The gene expression measured on day 0 of osteogenic differentiation was used as a calibrator to compare the gene expression of corresponding samples at other time intervals: Col I (D0), the calibrator for Col I hydrogels; Col I/II-HA (D0), the calibrator for Col I/II-HA hydrogels; Col I/II-HA+Cho (D0), the calibrator for Col I/II-HA+Cho hydrogels; Col I/II-HA+Cho_only (D0), the calibrator for Col I/II-HA+Cho_only hydrogels.

#### Alkaline phosphatase activity assay

Alkaline phosphatase (ALP) activity was measured on day 14 of the osteogenic differentiation in the DIF medium following the protocol described by Apinun et al.^32^ Briefly, the hydrogels containing the cells were removed from the Scaffdex holders, washed with PBS, and cut in half. One half of each sample was transferred to a fresh 24-well plate and minced into smaller pieces. Then, 0.5 mL of sodium dodecyl sulfate (SDS) was added to each well, and the hydrogels were incubated at 37°C in a 5% CO₂ atmosphere for 1 h to disrupt the cell membranes. After incubation, 20 µL of each sample was pipetted into a 96-well plate in triplicate, and 100 µL of p-nitrophenyl phosphate disodium salt (1-Step PNPP, Thermo Fisher Scientific, Waltham, MA, USA) was added to each well containing the cell lysates. The mixture was incubated at 37°C in a 5% CO₂ atmosphere for 15 min. The amount of p-nitrophenol, the product of p-nitrophenyl phosphate hydrolysis catalyzed by the ALP present in the samples, was measured using a VersaMax microplate spectrophotometer (Molecular Devices, San Jose, CA, USA) at an absorbance of 405 nm.

The other half of each sample was used for DNA extraction with TRI Reagent (Sigma-Aldrich, St. Louis, MO, USA). The samples were washed in PBS, placed in 2-mL tubes containing 1.4 mm ceramic homogenization beads (Qiagen, Hilden, Germany), and 1 mL of TRIzol. The tubes were then homogenized using a Precellys Evolution homogenizer (Bertin Instruments, Montigny-le-Bretonneux, France) at a speed of 1000 rpm for 30 s. DNA isolation was then carried out according to the manufacturer’s protocol. The amount of p-nitrophenol measured at 405 nm in each hydrogel was normalized to the DNA content (a.u./µg DNA).

#### Immunofluorescence staining of ALP

Immunofluorescence staining was used to evaluate ALP production in cells within hydrogels after 14 days. The staining procedure followed the protocol described by LeSavage et al.^33^ Briefly, the hydrogels were fixed in 4% paraformaldehyde (PFA) in PBS for 1 h at room temperature (RT). After fixation, the hydrogels were removed from the Scaffdex holders and transferred to a fresh 24-well plate. The hydrogels were then washed three times with PBS for 10 min each time. Then, permeabilization was performed using 750 µL of phosphate-b﻿uffered saline with tween-20 (PBST) (100 mL of PBS + 250 µL of Triton X-100) per sample for 1 h at RT on a rocker at 15 rpm. After aspirating the PBST, 750 µL of blocking solution (99.5 mL of PBS, 5 g of bovine serum albumin [BSA], and 0.5 mL of Triton X-100) was added to each hydrogel, and they were incubated for 3 h at RT on a rocker.

The primary antibody was diluted in an antibody solution (99.5 mL of PBS, 2.5 g of BSA, and 0.5 mL of Triton X-100) and added to the hydrogels. The plates were sealed with parafilm and incubated overnight at 4°C on a rocker. The primary antibody used was a mouse monoclonal anti-ALP antibody (1:200; Bio-Techne R&D Systems, Minneapolis, MN, USA).

The next day, the antibody solution was aspirated, and the samples were washed three times with PBST for 1 h at RT on a rocker. A secondary antibody, Alexa Fluor 546-conjugated F(ab’)2 fragment of goat anti-mouse IgG (1:400; Invitrogen, Thermo Fisher Scientific, Waltham, MA, USA), was added along with 4′,6-diamidino-2-phenylindole (DAPI, 1:500; Sigma-Aldrich, St. Louis, MO, USA) to stain cell nuclei. The hydrogels were incubated overnight at 4°C on a rocker and protected from light. Finally, the hydrogels were washed three times with 1 mL of PBST for 30 min at RT on a rocker and stored in PBS. Micrographs were captured using a spinning disk confocal system Dragonfly 503 (Oxford Instruments Andor, Belfast, UK) with a 10× objective and processed with Imaris 10.2 (Bitplain, Oxford Instruments Andor, Belfast, UK) and Fiji software.^34^ The micrographs are presented as maximum intensity projections (MIPs). Due to strong background interference, only a selected section of the acquired depth is shown, focusing on the area with the strongest signal and minimal background noise (MIP of approximately 40 µm).

#### Assessment of calcium deposition

Calcium deposition within hydrogels was assessed using the Calcium Colorimetric Assay Kit (Sigma-Aldrich, St. Louis, MO, USA). Briefly, the hydrogel constructs were removed from the Scaffdex holders, washed twice with PBS, and transferred to a fresh 24-well plate. One milliliter of SDS was added to each well, and the gels were minced with a spatula. Then, the gels were incubated at 37°C for 1 h on a shaker to disrupt the cell membranes. To quantify the calcium, 500 µL of the lysate was mixed with 1 mL of 0.5 M HCl and incubated overnight at 4°C on a shaker. The following day, the samples were equilibrated to RT in the dark and then centrifuged at 10,000 rpm for 10 min. Finally, 1 mL of the supernatant was collected. In a 96-well plate, 25 µL of each sample (or 12.5 µL sample + 12.5 µL 0.5 M HCl, if dilution was necessary) was combined with 45 µL of the chromogenic reagent and gently mixed, followed by the addition of 30 µL of calcium assay buffer and mixing again. The plates were then incubated for 5–10 min at RT in the dark and centrifuged at 300 rpm. After centrifugation, any bubbles were removed with a sterile needle. Absorbance was measured with Synergy HT microplate reader (Biotek, Winooski, VT, USA) at 575 nm within 30 min to avoid signal fading. Calcium concentrations were determined from a standard calibration curve.

#### Hydrogel unconfined compression test

The collagen hydrogels containing cells were stored in a humidified atmosphere at 37°C with 5% of CO_2_, pH ~ 7.4, and were measured under identical conditions. An unconfined compression test was performed using a customized inverted confocal microscope TCS SPE (Leica, Wetzlar, Germany). This microscope is equipped with an independent, movable, cylindrical platform. The platform includes a piezo force sensor Kistler 9217A (Kistler, Winterthur, Switzerland), which is connected to a Dewetron data acquisition system (Dewetron, Grambach, Austria). The other component of the setup is an objective holder that can be turned into an imaging position (10× objective) or a metal cylindrical platform. To normalize stress and strain, we measured the diameter of the gel disk (10.62 ± 0.46 mm; mean ± SD) using a reflectance confocal microscope, and the height (1.50 ± 0.22 mm; mean ± SD) using a calibrated platform distance and the moment when the force returned a non-zero value. To measure the force response and the viscous component, each sample was subjected to multiple strain steps at a speed of 100 µm/s. Each 15% deformation step was followed by a relaxation time period. The peak and relaxed forces were recalculated as stresses based on the geometry and then plotted against the strain values. The final values are represented as peak Young’s modulus (Pa) and relaxation Young’s modulus (Pa). The data were processed in Python (Python Software Foundation, Beaverton, OR, USA).

#### Histological analysis

Histological analysis was performed to observe cell migration from the PCL membrane and to characterize the newly deposited ECM in the hydrogels. First, the hydrogels were fixed in 4% PFA in PBS for 2 h at RT and then transferred to 70% ethanol before paraffin infusion using the standard protocol in the Leica HistoCore Arcadia H (Leica, Wetzlar, Germany). The paraffin-infused hydrogels were placed in a paraffin tissue block in the desired position and embedded using a paraffin embedding station Leica HistoCore Arcadia C (Leica, Wetzlar, Germany). The samples were sectioned using a Leica RM2255 microtome (Leica, Wetzlar, Germany), and the sections were dried at 42°C for 3 h.

Alcian blue staining was performed on rehydrated tissue sections according to a standardized protocol. Briefly, the slides were pretreated with 3% (v/v) acetic acid in deionized water for 3 min to acidify the tissue. Then, the slides were incubated for 30 min in a 1% (v/v) Alcian blue solution (Sigma-Aldrich, St. Louis, MO, USA) prepared in 3% (v/v) acetic acid adjusted to pH 1. The slides were rinsed with tap water and counterstained with Nuclear Fast Red (Sigma-Aldrich, St. Louis, MO, USA) for 10 min. After a final rinse in tap water, the sections were progressively dehydrated and then mounted using a Canadian balsam mounting medium (Sigma-Aldrich, St. Louis, MO, USA).

### Statistical analysis

Statistical analyses and graph generation were performed using GraphPad Prism 6 (GraphPad Software, Boston, MA, USA). The experimental data were analyzed using a two-way ANOVA or a one-way ANOVA followed by a Tukey’s multiple comparisons test. Comparisons were made separately for each experimental time interval. *P*-values < 0.05 were considered statistically significant.

## Results

We prepared *in vitro* models based on Col I hydrogels, either with or without Col II and HA, populated with different cell types (hMSCs, HUVECs, chondrocytes). A PCL nanofibrous membrane was used to support the hydrogels. It reduced the typical hydrogel shrinkage caused by cell traction forces and acted as a permeable barrier, allowing nutrients from the culture medium to diffuse underneath the model. The detailed composition of the hydrogel samples is described in Table 1. We cultivated these models in the osteogenic medium for up to 28 days without significant shrinkage to study cell migration and differentiation within them.

### Gene expression of osteogenic, chondrogenic, and hypertrophy markers

**Figure 3.**
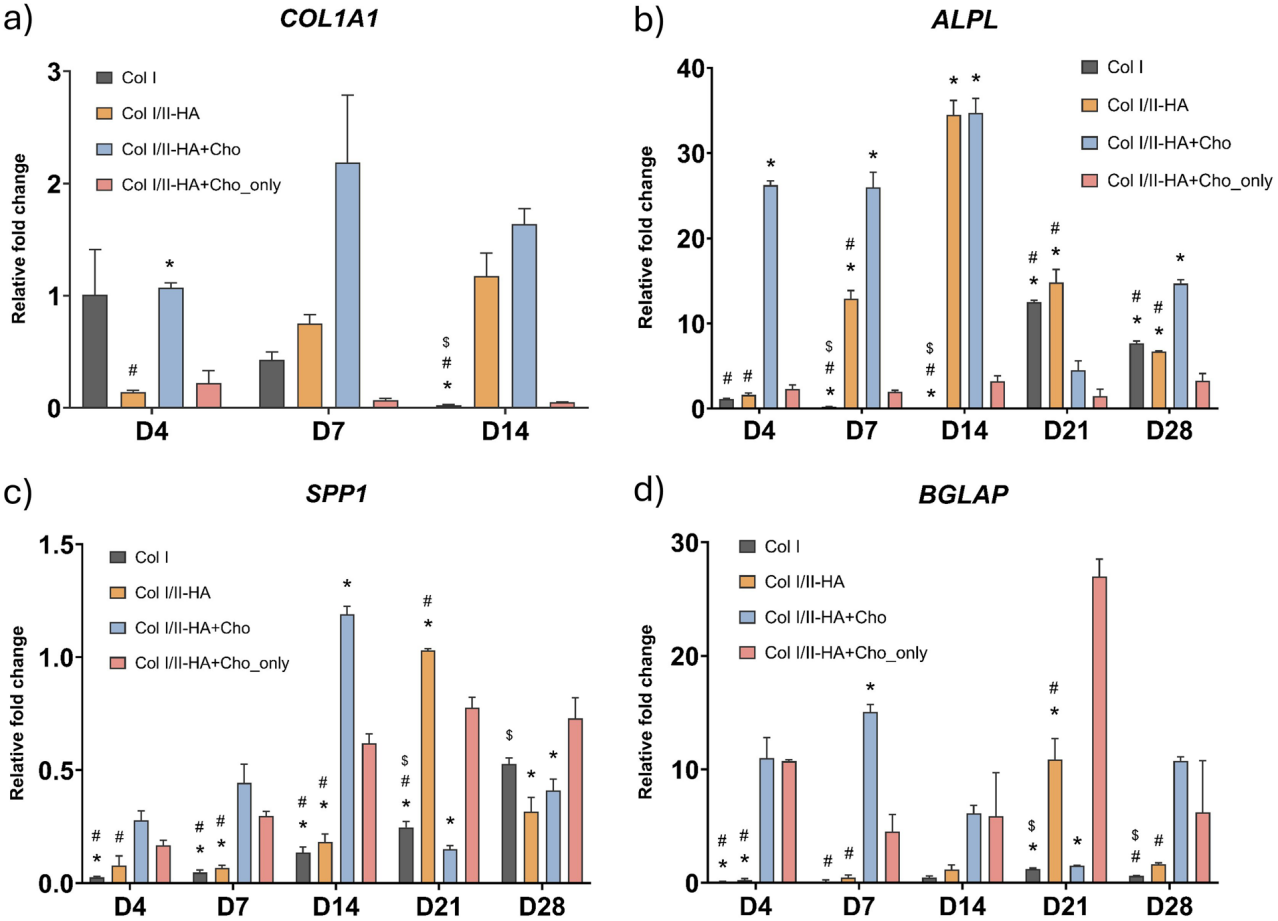
Relative expression of osteogenic genes during osteogenic differentiation: (a) collagen I (*COL1A1*) at days 4, 7, and 14; (b) alkaline phosphatase (*ALPL*), (c) osteopontin (*SPP1*), and (d) osteocalcin (*BGLAP*) at days 4, 7, 14, 21, and 28 in the studied hydrogels Col I, Col I/II-HA, Col I/II-HA+Cho, and Col I/II-HA+Cho_only. Mean of 2^−ΔΔCt^ + SD (n = 3). Expression compared to day 0 of the corresponding hydrogel, set as 1 (not shown on the graph). Two-way ANOVA with Tukey’s multiple comparisons test (*P* ≤ 0.05). Comparisons for each time interval separately; significance: $ in comparison with Col I/II-HA, # in comparison with Col I/II-HA+Cho, and * in comparison with Col I/II-HA+Cho_only.

**Figure 4.**
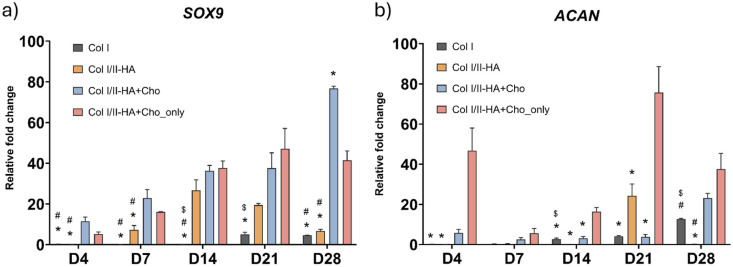
Relative expression of chondrogenic genes during osteogenic differentiation: (a) SRY-Box Transcription Factor 9 (*SOX9*) and (b) aggrecan (*ACAN*) at days 4, 7, 14, 21, and 28 in the studied hydrogels Col I, Col I/II-HA, Col I/II-HA+Cho, and Col I/II-HA+Cho_only. Mean of 2^−ΔΔCt^ + SD (n = 3). Expression compared to day 0 of the corresponding hydrogel, set as 1 (not shown on the graph). Two-way ANOVA with Tukey’s multiple comparisons test (*P* ≤ 0.05). Comparisons for each time interval separately; significance: $ in comparison with Col I/II-HA, # in comparison with Col I/II-HA+Cho, and * in comparison with Col I/II-HA+Cho_only.

**Figure 5.**
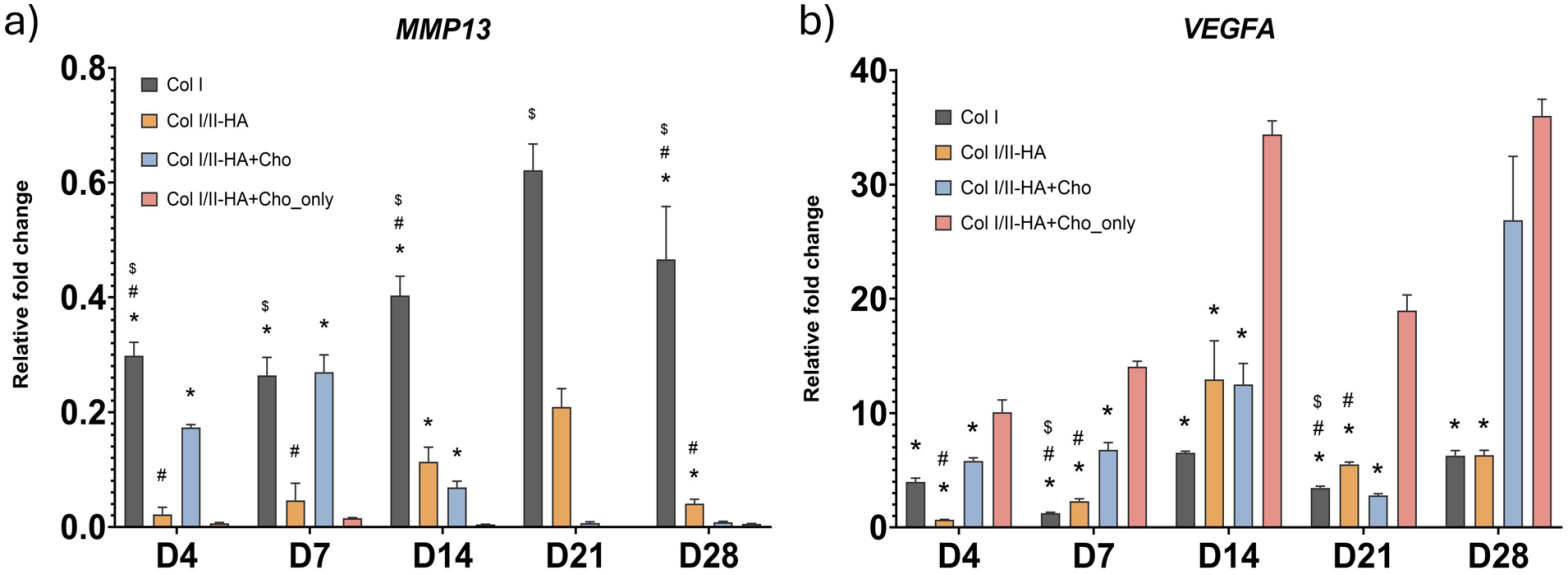
Relative expression of hypertrophy-associated genes during osteogenic differentiation: (a) matrix metallopeptidase 13 (*MMP13*) and (b) vascular endothelial growth factor A (*VEGFA*) at days 4, 7, 14, 21, and 28 in the studied hydrogels Col I, Col I/II-HA, Col I/II-HA+Cho, and Col I/II-HA+Cho_only. Mean of 2^−ΔΔCt^ + SD (n = 3). Expression compared to day 0 of the corresponding hydrogel, set as 1 (not shown on the graph). Two-way ANOVA with Tukey’s multiple comparisons test (*P* ≤ 0.05). Comparisons for each time interval separately; significance: $ in comparison with Col I/II-HA, # in comparison with Col I/II-HA+Cho, and * in comparison with Col I/II-HA+Cho_only.

The gene expression of the following markers was measured by qPCR at predetermined time intervals (0, 4, 7, 14, 21, and 28 days) after the first addition of osteogenic medium to assess the osteogenic and chondrogenic differentiation of cells as well as the expression of markers of chondrocyte hypertrophy in all hydrogel models: Col I alpha-1 chain (*COL1A1*), alkaline phosphatase (*ALPL*), osteopontin (*SPP1*), osteocalcin (*BGLAP*; Fig. 3), SRY-box transcription factor 9 (*SOX9*), aggrecan (*ACAN*; Fig. 4), matrix metallopeptidase 13 (*MMP13*) and vascular endothelial growth factor A (*VEGFA*; Fig. 5).

*COL1A1* is considered an early osteogenic marker, so its expression was studied during the first 14 days of the osteogenic differentiation (Fig. 3a). During this period, distinct expression patterns were observed among the different hydrogels. Cells in the Col I hydrogel and the Col I/II-HA+Cho_only hydrogel exhibited a gradual decline in *COL1A1* expression. In contrast, cells in the Col I/II-HA hydrogel exhibited an initial drop by day 4, followed by recovery by day 14. The most pronounced increase in *COL1A1* expression occurred in the Col I/II-HA+Cho hydrogel, peaking on day 7.

Statistical analysis revealed no significant difference in *COL1A1* expression between the Col I and Col I/II-HA hydrogels on days 4 and 7. However, after 14 days of osteogenic differentiation, the *COL1A1* expression was significantly higher in the Col I/II-HA. Furthermore, the presence of chondrocytes in the Col I/II-HA+Cho hydrogel enhanced *COL1A1* expression more than the Col I/II-HA hydrogel alone, suggesting that chondrocytes may influence *COL1A1* expression.

*ALPL*, an early-to-middle osteogenic marker, was analyzed throughout the entire osteogenic cultivation period (Fig. 3b). Expression patterns varied across different hydrogels. In the Col I hydrogel, *ALPL* expression initially decreased on days 7 and 14, then increased again by day 21. In contrast, *ALPL* expression in the Col I/II-HA hydrogel gradually increased, peaking on day 14, and then declined steadily until day 28. The presence of chondrocytes in the Col I/II-HA+Cho hydrogel further enhanced *ALPL* expression, with an early rise on day 4, a peak on day 14, a sudden drop on day 21, and another increase on day 28. As expected, the lowest *ALPL* expression was observed in the Col I/II-HA-Cho_only hydrogel, which contained only chondrocytes.

Statistical analysis revealed that *ALPL* expression was significantly higher in cells within the Col I/II-HA hydrogel than in those within the Col I hydrogel during the middle stages of osteogenic differentiation (days 7 and 14), although levels equalized in the later stages (days 21 and 28). The presence of chondrocytes in the Col I/II-HA+Cho hydrogel led to a significantly faster increase in *ALPL* expression during the early phase (days 4 and 7) compared to the Col I/II-HA hydrogel. However, by day 14, both hydrogels reached a similar peak, which declined by day 21. Notably, *ALPL* expression in the Col I/II-HA+Cho hydrogel began to rise again by day 28, while it continued to decrease in the Col I/II-HA hydrogel.

*SPP1*, a middle-to-late osteogenic marker, was analyzed throughout the entire osteogenic cultivation period (Fig. 3c). An initial drop in expression was observed across all four hydrogels compared to day 0 of osteogenic differentiation. In the Col I hydrogel, *SPP1* expression increased steadily from day 4 to the end of the cultivation period. In contrast, the expression was not that stable in the Col I/II-HA hydrogel. After a slight dip on day 7, *SPP1* expression increased and peaked on day 21, followed by a decrease on day 28. Cells in the Col I/II-HA+Cho hydrogel exhibited a comparable pattern with an earlier peak on day 14, a decline on day 21, and a renewed increase on day 28. Notably, *SPP1* expression was also detected in the Col I/II-HA+Cho_only hydrogel containing only chondrocytes, suggesting that the chondrocytes themselves directly contribute to its expression.

Statistical analysis revealed no significant differences in *SPP1* expression between the Col I and Col I/II-HA hydrogels during the early and middle stages of osteogenic differentiation (days 4, 7, and 14). However, *SPP1* expression increased sharply in the Col I/II–HA hydrogel on day 21, while only a gradual increase was observed in the Col I hydrogel. Adding chondrocytes to the Col I/II-HA+Cho hydrogel significantly increased *SPP1* expression compared to the Col I/II-HA hydrogel. While both hydrogels exhibited a peak followed by a decline, the presence of chondrocytes led to an earlier and more pronounced peak, suggesting either a synergistic effect between cell types or a direct contribution of chondrocytes to *SPP1* expression.

*BGLAP*, a late osteogenic marker, was analyzed throughout the entire osteogenic cultivation period (Fig. 3d). The lowest levels were observed in the Col I hydrogel, where expression gradually increased until day 21, followed by a decline on day 28. A similar trend was observed in the Col I/II-HA hydrogel, with a more pronounced peak on day 21, followed by a decline on day 28. In contrast, the hydrogels containing chondrocytes exhibited different trends, with an earlier rise in expression (day 4). In the Col I/II-HA+Cho hydrogel, expression peaked on day 7, followed by a decline until day 21, and then increased on day 28. Interestingly, the expression in the Col I/II-HA+Cho_only hydrogel increased early, declined by day 7, peaked by day 21, and then decreased by day 28.

Statistical analysis revealed no significant difference in *BGLAP* expression between the Col I and Col I/II-HA hydrogels during the early and middle stages of osteogenic differentiation (days 4, 7, and 14). The expression followed a similar pattern in both hydrogels, gradually increasing and peaking on day 21, followed by a decline on day 28. However, the Col I/II-HA hydrogel showed a significantly higher spike in expression on day 21 than the Col I hydrogel. The presence of chondrocytes in the Col I/II-HA+Cho hydrogel significantly enhanced *BGLAP* expression at the early (days 4 and 7) and late (day 28) time points compared to the Col I/II-HA hydrogel. Notably, the relatively high early *BGLAP* expression in the Col I/II-HA+Cho_only hydrogel, along with its peak on day 21, suggests that chondrocytes alone may directly contribute to *BGLAP* expression.

*SOX9*, an early chondrogenic marker, was analyzed throughout the entire cultivation period (Fig. 4a). In cells within the Col I hydrogel, *SOX9* expression initially decreased compared to day 0 of osteogenic differentiation but increased slightly at later time points (days 21 and 28). Similarly, *SOX9* expression decreased in the Col I/II-HA hydrogel on day 4 and began to rise on day 7, peaking on day 14 before gradually declining until day 28. The addition of chondrocytes to both the Col I/II-HA+Cho hydrogel and the Col I/II-HA+Cho_only hydrogel led to an early increase in *SOX9* expression on day 4. The Col I/II-HA+Cho hydrogel showed a gradual rise, peaking on day 28. In contrast, the Col I/II-HA+Cho_only hydrogel reached its peak on day 21 before decreasing on day 28.

Statistical analysis revealed no significant difference in *SOX9* expression between the Col I and Col I/II-HA hydrogels during the early stages of chondrogenic differentiation (days 4 and 7). However, *SOX9* expression significantly increased on day 14 in the Col I/II-HA hydrogel, while it remained low in the Col I hydrogel until day 21. Notably, the presence of chondrocytes in the Col I/II-HA+Cho and Col I/II-HA+Cho_only hydrogels significantly enhanced *SOX9* expression, particularly during the early stages (days 4 and 7) and on day 28, compared to the Col I/II-HA hydrogel.

*ACAN*, a late marker of chondrogenic differentiation, exhibited distinct expression patterns across the hydrogels (Fig. 4b). In cells within the Col I hydrogel, *ACAN* expression initially decreased compared to day 0 of osteogenic differentiation and remained low until day 14, gradually increasing until day 28. A similar trend was observed in the Col I/II-HA hydrogel, though it exhibited a sudden peak on day 21, followed by a decline on day 28. Hydrogels containing chondrocytes showed an early increase on day 4, but with different trajectories. In the Col I/II-HA+Cho hydrogel, expression initially increased, then decreased on day 7, and subsequently rose steadily, culminating in a spike on day 28. In contrast, the Col I/II-HA+Cho_only hydrogel exhibited a much stronger initial increase on day 4, followed by a sharp decline and then a gradual increase, reaching its highest peak on day 21 before declining on day 28.

Statistical analysis revealed no significant difference in *ACAN* expression between the Col I and Col I/II-HA hydrogels during the early stages of chondrogenic differentiation (days 4 and 7). However, while the Col I hydrogel showed a steady increase from day 14 onward, expression in the Col I/II-HA hydrogel was more variable, peaking at day 21 before dropping at day 28. Notably, the Col I/II-HA+Cho_only hydrogel, which contained only chondrocytes, exhibited significantly higher *ACAN* expression than the other hydrogels, especially on day 21. Interestingly, in the Col I/II-HA+Cho hydrogel containing hMSCs, HUVECs, and chondrocytes, this enhancement was delayed and became evident only on day 28.

*MMP13*, a marker of chondrocyte hypertrophy, was analyzed throughout the entire cultivation period (Fig. 5a). Compared to day 0 of osteogenic differentiation, a decrease in expression was observed across all four hydrogels (<1). In the Col I hydrogel, expression levels were relatively low on days 4 and 7. Then, they increased steadily, peaking on day 21, before slightly decreasing by day 28. A similar trend was observed in the Col I/II-HA hydrogel, with expression increasing from day 4 to a peak on day 21, followed by a decline on day 28. In the presence of chondrocytes within the Col I/II-HA+Cho hydrogel, the *MMP13* expression was higher at early time points (days 4 and 7) but progressively declined after day 14. Expression levels were low by day 21 and remained minimal by day 28. In contrast, the Col I/II-HA+Cho_only hydrogel, which contained only chondrocytes, exhibited consistently low expression levels at all time points.

Statistical analysis revealed significantly lower expression in the Col I/II-HA hydrogel than in the Col I hydrogel at all time points. Including chondrocytes in the Col I/II-HA+Cho hydrogel led to a significant increase in *MMP13* expression during the early stages of osteogenic differentiation (days 4 and 7), compared to the Col I/II-HA hydrogel. However, as expression levels in the Col I/II-HA+Cho hydrogel declined over time, *MMP13* expression in the Col I/II-HA hydrogel became significantly higher by day 28.

*VEGFA*, a marker associated with both chondrocyte hypertrophy and angiogenesis, was analyzed throughout the entire cultivation period (Fig. 5b). *VEGFA* expression varied substantially between hydrogel types and over time. In the Col I hydrogel, expression increased moderately on day 4, decreased on day 7, and increased again on day 14. Expression decreased by day 21, then increased again by day 28. A similar trend was observed in the Col I/II-HA hydrogel, where expression was lower on day 4, increased steadily through day 14, declined on day 21, and increased slightly by day 28. Including chondrocytes in the Col I/II-HA+Cho and Col I/II-HA+Cho_only hydrogels resulted in early upregulation of *VEGFA* expression on days 4 and 7. The expression pattern was similar in both hydrogels, with an initial peak on day 14, a decline on day 21, and a second, more pronounced peak on day 28.

Statistical analysis revealed that *VEGFA* expression was significantly higher in the Col I/II-HA hydrogel than in the Col I hydrogel on days 7 and 21 of osteogenic differentiation; however, this difference disappeared by day 28. The presence of chondrocytes in the Col I/II-HA+Cho hydrogel significantly increased *VEGFA* expression at the early time points (days 4 and 7) compared to the Col I/II-HA hydrogel. However, this difference was no longer apparent by day 14. Notably, chondrocytes alone exhibited significantly higher *VEGFA* expression in the Col I/II-HA+Cho_only hydrogel than in all other hydrogels at all time points. However, this difference was no longer evident in the Col I/II-HA+Cho hydrogel by day 28.

### Activity of alkaline phosphatase

**Figure 6.**
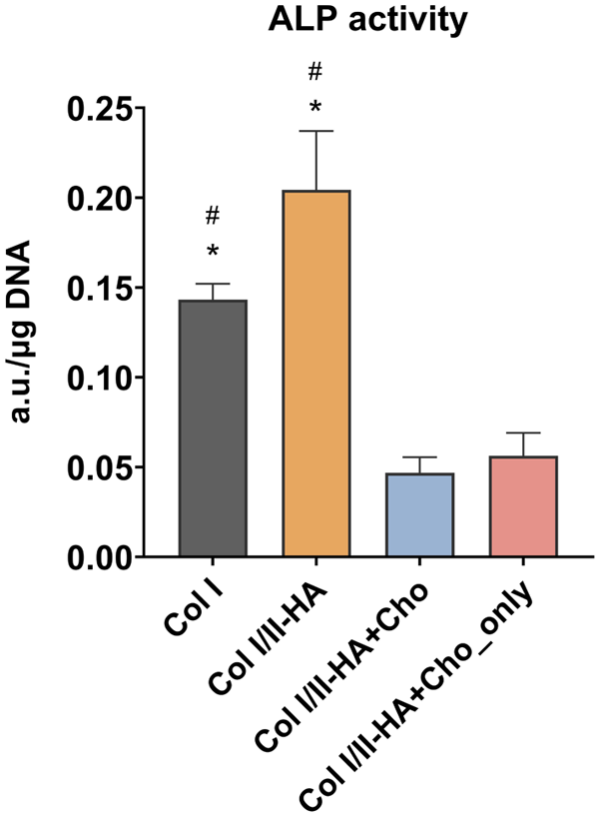
ALP enzyme activity in the studied hydrogels Col I, Col I/II-HA, Col I/II-HA+Cho, and Col I/II-HA+Cho_only after 14 days of osteogenic differentiation in the DIF medium based on a colorimetric assay measurement. The data are expressed as absorbance at 405 nm (a.u.)/µg DNA + SD (n = 3). Significance was assessed with one-way ANOVA with Tukey’s multiple comparisons test (*P* ≤ 0.05) and is shown as $ in comparison with Col I/II-HA, # in comparison with Col I/II-HA+Cho, and * in comparison with Col I/II-HA+Cho_only.

The enzymatic activity of ALP was measured on day 14 of osteogenic differentiation using a biochemical assay (Fig. 6). ALP activity appeared slightly higher in the Col I/II-HA hydrogel, which contains Col II and HA, compared to the Col I hydrogel; however, this difference was not statistically significant. In contrast, the inclusion of chondrocytes in the Col I/II-HA+Cho and Col I/II-HA+Cho_only hydrogels led to a significant reduction in ALP activity compared to hydrogels without chondrocytes.

### Production of ALP protein

**Figure 7.**
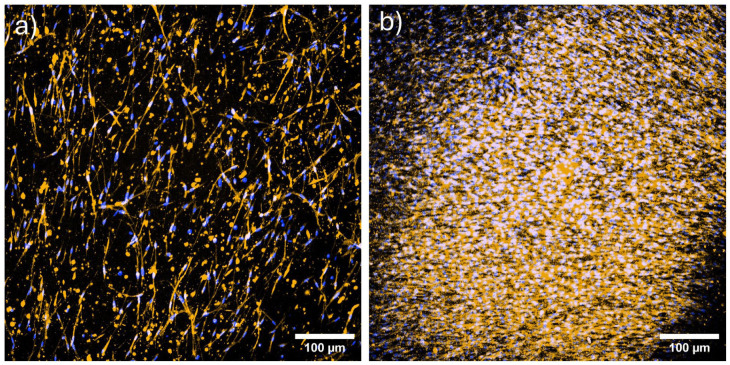
Maximum intensity projection (MIP) of ALP (red) and cell nuclei (blue) in (a) Col I/II-HA hydrogel and (b) Co I/II-HA+Cho hydrogel after 14 days of osteogenic differentiation in the DIF medium. MIP of 82 slices (approximately 40 µm). Spinning disk confocal microscope (Andor Dragonfly 503). 10× objective. Scale bar = 100 µm.

The production of ALP in Col I/II-HA and Col I/II-HA+Cho hydrogels was visualized using immunofluorescence after 14 days of osteogenic differentiation and presented as a maximum-intensity projection of 82 slices (Fig. 7). As expected, fewer cells were found in the Col I/II-HA hydrogel (Fig. 7a), as it contained only hMSCs and HUVECs that had migrated from the PCL membrane. In contrast, the Col I/II-HA+Cho hydrogel (Fig. 7b) had a higher cell density, as it not only contained migrated hMSCs and HUVECs from co-culture but also chondrocytes, which were added directly to the hydrogel solution before polymerization. The ALP staining appeared to be stronger in the Col I/II-HA+Cho hydrogel (Fig. 7b).

### Calcium deposition

**Figure 8.**
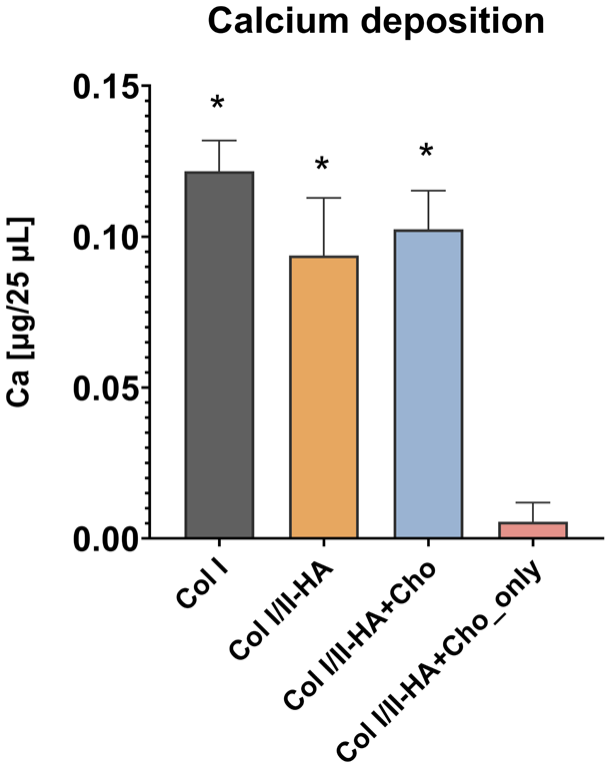
Calcium deposition within the hydrogels Col I, Col I/II-HA, Col I/II-HA+Cho, and Col I/II-HA+Cho_only after 28 days of osteogenic differentiation in the DIF medium based on a colorimetric assay measurement. The data are expressed as µg calcium/25 µL + SD (n = 3). Significance was assessed with one-way ANOVA with Tukey’s multiple comparisons test (*P* ≤ 0.05) and is shown as $ in comparison with Col I/II-HA, # in comparison with Col I/II-HA+Cho, and * in comparison with Col I/II-HA+Cho_only.

Calcium deposition was measured on day 28 of osteogenic differentiation using the Calcium Colorimetric Assay Kit (Fig. 8). Calcium deposition was significantly higher in the hydrogels containing the hMSCs/HUVECs co-culture than in the hydrogel containing only chondrocytes (Col I/II-HA+Cho_only). However, no significant difference in calcium deposition was observed between the Col I, Col I/II-HA, and Col I/II-HA+Cho hydrogels.

### Histological analysis

**Figure 9.**
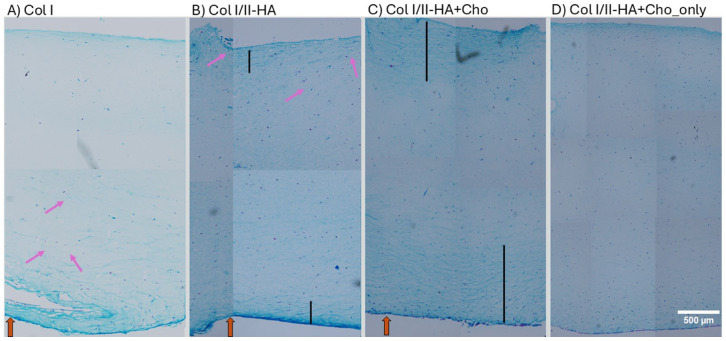
Histological characterization of Col I (a), Col I/II-HA (b), Col I/II-HA+Cho (c), and Col I/II-HA+Cho_only (d) hydrogels after 28 days of osteogenic differentiation in the DIF medium. Alcian blue staining. Thick orange arrows indicate the bottom side of the hydrogel and the direction of cell migration from the PCL membrane (a, b, and c). Hydrogel D contained only chondrocytes; therefore, no hMSCs/HUVECs migrated from the bottom. Pink, thinner arrows indicate cells that migrated from the bottom. Black lines in b and c indicate the thickness of stronger Alcian blue staining. Scale bar = 500 µm.

Alcian blue staining of histological sections was performed to evaluate glycosaminoglycan (GAG) distribution and demonstrate cell migration and distribution in all types of hydrogels after 28 days of osteogenic differentiation. By this time point, the hMSCs/HUVECs that were seeded on the PCL membrane had migrated into the Col I, Col I/II-HA, and Col I/II-HA+Cho hydrogels (Fig. 9). The Col I/II-HA+Cho_only hydrogel contained only chondrocytes; therefore, we did not observe any cell migration from the bottom. Instead, the chondrocytes were evenly distributed throughout the hydrogel. In the Col I hydrogel, most cells remained concentrated at the bottom of the gel or migrated a short distance (Fig. 9A). However, in the Col I/II-HA hydrogel, cells migrated from the bottom toward the top (Fig. 9B). This suggests that the Col II and HA in the Col I/II-HA hydrogel facilitated cell migration. In the Col I/II-HA+Cho hydrogel, cells were also present in the top section. However, since this hydrogel contains chondrocytes, we cannot determine whether these cells migrated from the bottom or if they are the original chondrocytes present in the hydrogel. The most homogeneous cell distribution was observed in the Col I/II-HA+Cho_only hydrogel (Fig. 9D) due to the lack of hMSCs/HUVECs co-culture at the bottom.

The most intense Alcian blue staining was observed in the Col I hydrogel at the bottom, where most of the cells were located. We observed more intense Alcian blue staining in the Col I/II-HA and Col I/II-HA+Cho hydrogels than in the Col I hydrogel. Additionally, staining was seen not only at the bottom of these two hydrogels but also at the top, indicating further deposition of the cartilaginous matrix. In the Col I/II-HA+Cho hydrogel containing chondrocytes, the staining penetrated deeper into the hydrogel from both sides (Fig. 9C, indicated by the black line), compared to the Col I/II-HA hydrogel lacking chondrocytes. Interestingly, the Col I/II-HA+Cho_only hydrogel containing only chondrocytes showed weak Alcian blue staining. These findings suggest that combining hMSCs and chondrocytes results in greater cartilage matrix deposition, indicating a synergistic effect between the two cell types.

### Mechanical properties of hydrogels

We performed an unconfined compression test to assess the mechanical properties of each hydrogel type, measuring the Young’s modulus at peak and during relaxation. Young’s modulus at peak reflects both the viscose and elastic components of the gels. Over time, the viscous component relaxes, causing the material response to shift to the elastic component, which is reflected in the Young’s modulus at relaxation. The results are summarized in Table 3.

**Table 3.** Young’s modulus at peak (viscoelastic response) and at relaxation (elastic response) for gels.

Day	Group	N (peak)	Peak modulus (Pa) mean	Peak modulus (Pa) std	N (relax)	Relaxation modulus (Pa) mean	Relaxation modulus (Pa) std
d0	Col I/II-HA	3	5457	1296	3	835	240
d0	Col I/II-HA+Cho	2	3606	158	2	474	76
d0	Col I/II-HA+Cho_only	3	4291	1744	3	655	227
d0	Col I	3	15,398	6575	3	992	170
d28	Col I/II-HA	3	4910	2658	3	655	85
d28	Col I/II-HA+Cho	3	10,738	4511	3	490	295
d28	Col I/II-HA+Cho_only	2	10,388	193	2	675	114
d28	Col I	3	4279	559	3	509	16

## Discussion

This study investigated the impact of hydrogel composition and the presence of chondrocytes on the osteogenic differentiation of hMSCs co-cultured with HUVECs, aiming to simulate the early stages of EO. Understanding this process is important not only for establishing an appropriate *in vitro* model that can be used for further developmental, (patho)physiological, pharmacological, and other studies but also for limiting or replacing the use of laboratory animals in modern science according to the 3Rs principle. It is also relevant for regenerative medicine, including bone tissue engineering. Currently, this primarily relies on intramembranous ossification, where osteoblast precursors, such as bone marrow–derived MSCs, differentiate directly into osteoblasts. However, this approach bypasses the cartilaginous template that is physiologically relevant for the formation of most bones in the human body^35^. Our findings emphasize the significant roles of Col II and HA in modulating osteogenic differentiation and the influence of chondrocytes in shaping cell differentiation.

### Influence of Col II and HA on osteogenic differentiation

The presence of Col II and HA in the Col I/II-HA hydrogel significantly affected the expression patterns of key osteogenic markers. Unlike the pure Col I hydrogel, where *COL1A1* expression (Fig. 3a) gradually declined over time, the Col I/II-HA hydrogel steadily increased, peaking on day 14 of osteogenic differentiation. Similar trends were observed in *ALPL* expression (Fig. 3b), a marker of mid-stage osteogenic differentiation. The Col I/II-HA hydrogel promoted a stronger increase during the mid-stages of differentiation (days 7 and 14) than the Col I hydrogel did, peaking on day 14 before subsequently declining. While the Col I/II-HA hydrogel, containing Col II and HA, showed a slight increase in ALP enzymatic activity on day 14 compared to the Col I hydrogel (Fig. 6), this difference was not statistically significant. These findings suggest that although Col II and HA may provide biochemical cues supportive of early osteogenic differentiation, their effect on ALP activity at this mid-stage is modest under the current experimental conditions. Nonetheless, previous studies have highlighted the potential of these matrix components in promoting osteogenesis. For example, Chiu et al.^36^ demonstrated that Col II-coated surfaces enhanced calcium deposition and upregulated the mRNA expression of osteogenic markers, such as *RUNX2*, osteocalcin, and *ALPL*, in hMSCs, likely through activation of the integrin α2β1-FAK-JNK signaling axis^37^. Similarly, HA has been reported to stimulate the proliferation of osteoprogenitor cells and enhance the expression of genes encoding bone matrix proteins, and HA-based hydrogels have supported osteogenic differentiation in various *in vitro* models^38–43^. Together, these observations suggest that although Col II and HA are known to support osteogenic differentiation, their influence on ALP activity in this model was limited, indicating that other factors, such as cell-cell interactions or culture conditions, may play a more dominant role under the present experimental setup.

In the Col I and Col I/II-HA hydrogels, the expression of late-stage osteogenic markers *SPP1* (Fig. 3c) and *BGLAP* (Fig. 3d) initially decreased compared to day 0 of osteogenic differentiation, followed by a gradual increase over time. This early decline may be linked to enhanced cell migration during the initial time intervals, which temporarily slows differentiation^44^. However, *SPP1* and *BGLAP* exhibited higher expression peaks in the Col I/II-HA hydrogel than in the Col I hydrogel. In the Col I hydrogel, *SPP1* reached its peak on day 28, while *BGLAP* peaked on day 21, both following a gradual increase. In contrast, both markers rose steadily in the Col I/II-HA hydrogel, peaking sharply on day 21 at significantly higher levels before declining by day 28. These results suggest that Col II and HA enhance not only early- and mid-stage differentiation, as discussed earlier, but also promote late-stage osteogenic maturation. The observed drop in *SPP1* and *BGLAP* expression after day 21 in the Col I/II-HA hydrogel may be due to regulatory feedback mechanisms that downregulate mRNA expression once sufficient levels of osteogenic proteins have been synthesized. Osteogenic differentiation is well-known to be tightly regulated through signaling pathways, such as Wnt/β-catenin and BMP/TGF-β pathways, in which negative feedback loops play a role in maintaining homeostasis^45,46^. Additionally, the decline in mRNA levels may coincide with increased protein stability, meaning that transcriptional activity is reduced once adequate protein levels are reached^47,48^. Interestingly, the impact of HA on cellular proliferation and osteogenic differentiation *in vitro* appears to be highly dependent on its molecular weight (MW) and concentration^42^. In our study, we used high-MW HA (>1000 kDa), which has been shown to significantly increase osteocalcin mRNA expression, mineralization, and ALP activity in rat calvarial cell cultures in a concentration-dependent manner^49^. However, its effects vary across different cell types. While some studies report no effect of high-MW HA on bone-related gene expression in periodontal ligament cells^40^, others suggest that it can inhibit osteogenic differentiation in both mouse myoblastic and mesenchymal cells^41^. Nevertheless, the results of our study suggest that HA, when combined with Col I and Col II at a concentration of 2.5 mg/mL hydrogel, does not inhibit osteogenic differentiation. Calcium deposition was detected in both Col I and Col I/II-HA hydrogels (Fig. 8); however, no significant difference was observed between them. This indicates that Col II and HA, at the tested concentrations, did not substantially affect calcium deposition by the cells.

Overall, these findings suggest that including Col II and HA in the composite hydrogel modestly enhances osteogenic differentiation. While their effects were most apparent during the early stage and mid-stage of differentiation, they also contributed to more pronounced expression of late-stage osteogenic markers. Although calcium deposition was not significantly affected, these matrix components appear to provide supportive biochemical cues that sustain osteogenic progression. This implies their potential value in osteochondral tissue engineering.

### Influence of chondrocytes on osteogenic differentiation

Adding chondrocytes to the Col I/II-HA+Cho hydrogel further enhanced osteogenic differentiation compared to Col I/II-HA alone. The presence of chondrocytes resulted in increased *COL1A1* expression (Fig. 3a), likely due to paracrine signaling, whereby chondrocytes secrete specific growth factors or morphogens that influence neighboring cells^50^. One such factor is TGF-β, which is known to regulate ECM production^51^ and induce Col I expression^52^. Similarly, *ALPL* expression (Fig. 3b) increased more rapidly in the Col I/II-HA+Cho hydrogel than in the Col I/II-HA hydrogel during the early stages, suggesting that chondrocytes may accelerate the initial differentiation process. This finding aligns with the results of Thompson et al.^53^, who observed increased *ALPL* expression in hMSCs co-cultured with chondrocytes measured on days 8 and 14. Interestingly, chondrocytes cultured alone in the Col I/II-HA+Cho_only hydrogel exhibited relatively low expression levels of both *COL1A1* and *ALPL*, suggesting that their upregulation depends on synergistic interactions with other cell types rather than the direct contribution by the chondrocytes themselves. Furthermore, the immunofluorescence staining of the ALP protein on day 14 of osteogenic differentiation (Fig. 7) appeared to be stronger in the Col I/II-HA+Cho hydrogel than in the Col I/II-HA hydrogel. This suggests a higher ALP protein content in the presence of chondrocytes. This suggests that chondrocytes either enhance the protein production through a synergistic effect on hMSCs^54^ or are undergoing hypertrophy, which is a normal transient state during EO, and are producing the ALP themselves^55^. In contrast, the enzymatic activity measured on day 14 (Fig. 6) was significantly higher in the Col I/II-HA hydrogel than in the Col I/II-HA+Cho hydrogel, suggesting that the ALP activity was inhibited in the presence of chondrocytes. ALP catalyzes the hydrolysis of phosphate groups, increasing the local concentration of inorganic phosphate^56^, which is known to be a competitive inhibitor of the ALP^57^. Consequently, the elevated ALP content suggested by the stronger staining in the Col I/II-HA+Cho hydrogel (Fig. 7b) may have led to higher inorganic phosphate levels, resulting in feedback suppression of ALP activity. This observation aligns with previous studies showing reduced ALP activity in co-cultures of hMSCs and articular chondrocytes under chondrogenic conditions^58^. Another potential explanation is that ALP may undergo post-translational modifications or exist in an inactive state in the presence of chondrocytes. ALP is a glycosylated enzyme, and its activation can be influenced by various factors, including proteolytic processing, phosphorylation, glycosylation, or dimerization^59^. The chondrocyte-rich environment may influence these modifications, potentially delaying ALP activation or trapping it within the ECM in an inactive form. Furthermore, chondrocytes produce Col II, proteoglycans, and other ECM molecules that may interact with ALP by binding to it or altering its conformation. This could affect its localization and activation, thereby preventing optimal enzymatic function despite higher protein levels^60^. By day 28, *ALPL* expression increased again in the Col I/II-HA+Cho hydrogel, whereas it continued to decline in the Col I/II-HA hydrogel. This may indicate prolonged osteogenic activity due to the chondrocyte-mediated microenvironment.

The expression patterns of *SPP1* (Fig. 3c) and *BGLAP* (Fig. 3d) in hydrogels containing chondrocytes further highlight their role in osteogenic differentiation. The early and pronounced *SPP1* peak in the Col I/II-HA+Cho hydrogel points to a synergistic interaction between chondrogenic and osteogenic pathways. Similarly, the dynamic fluctuations of *BGLAP* expression, with an early peak on day 7, followed by a decline and subsequent increase, suggest a complex regulation of late-stage osteogenesis influenced by chondrocytes. Notably, *SPP1* and *BGLAP* were also detected in the Col I/II-HA+Cho_only hydrogel, which contained only chondrocytes. This finding suggests that chondrocytes may directly contribute to the expression of these osteogenic markers, supporting the hypothesis that they actively participate in osteogenic processes. The relatively low *BGLAP* levels observed in hydrogels lacking chondrocytes further reinforce this notion, potentially reflecting limited hMSC differentiation due to prolonged migration or insufficient differentiation cues, despite exposure to osteogenic medium. In these cells, progression toward differentiation is accompanied by a reduction in proliferation, reflecting a shift into the G₀ phase of the cell cycle triggered by adequate biochemical cues^61^. Such stimuli may include osteogenic molecules secreted by chondrocytes, such as osteocalcin^62^. Consistent with this, chondrocytes in the Col I/II-HA+Cho and Col I/II-HA+Cho_only hydrogels expressed *BGLAP* from the beginning, indicating their early involvement in osteogenic processes. Conversely, the calcium deposition assay (Fig. 8) revealed that, compared to the other hydrogels containing a co-culture of HUVECs and hMSCs, chondrocytes alone deposit significantly lower amounts of calcium.

Chondrocytes can be involved in the process of EO in two ways. One way is the indirect pathway, which includes the terminal differentiation of chondrocytes, manifested by hypertrophy and programmed cell death. This process promotes vascular invasion, allowing osteoblast precursors to migrate into the area. The other way is the direct pathway, which involves the transition of hypertrophic chondrocytes to osteoblasts^35^. It is difficult to determine which pathway played a greater role in our study; however, the direct pathway is suggested by the highest expression of *BGLAP* in the Col I/II-HA+Cho_only hydrogels containing only chondrocytes.

Overall, these findings emphasize the crucial role of chondrocytes in osteogenic differentiation. This role may be due to their secretion of osteoinductive signals and contribution to ECM remodeling. However, the possibility of their direct transition to osteoblasts should be further elucidated. Nevertheless, this study highlights the potential of chondrocytes as key players in bone formation, facilitating the transition from cartilage to bone during the process of osteogenesis^50^.

### Chondrogenic and hypertrophy marker expression and their implications

The expression patterns of the chondrogenic markers *SOX9* (Fig. 4a) and *ACAN* (Fig. 4b) further demonstrate the interplay between osteogenesis and chondrogenesis. *SOX9*, a key transcription factor, governs chondrocyte differentiation and is essential for maintaining the chondrogenic phenotype^63^. The late appearance of *SOX9* expression in Col I hydrogel, detectable only at day 21, indicates a delayed or minimal activation of chondrogenic transcriptional programs. This delay likely reflects the absence of cartilage-specific ECM components (e.g., Col II and HA) that normally promote early *SOX9* activity and mesenchymal condensation^64,65^. Building on this, *SOX9* expression increased significantly in the Col I/II-HA hydrogel, peaking on day 14, suggesting that Col II and HA promote a chondrogenic environment during early stages of differentiation. This observation is consistent with previous reports that MSCs upregulate *SOX9* expression during mesenchymal condensation, which is a crucial step during the EO^6^. Furthermore, numerous studies have demonstrated that Col II-based scaffolds not only help maintain the chondrocyte phenotype but also enhance the chondrogenic differentiation of MSCs^66^. In addition, both Col II and HA have been consistently reported to exhibit inherent chondrogenic potential^67^. The decline in *SOX9* after day 14 suggests a shift toward osteogenic and endothelial maturation as matrix organization progresses. The presence of chondrocytes in the Col I/II-HA+Cho and Col I/II-HA+Cho_only hydrogels further amplified *SOX9* expression, particularly in the early and late stages, indicating that chondrocytes actively contribute to maintaining a chondrogenic niche within the hydrogel.

Similarly, *ACAN* gene expression followed distinct trends (Fig. 4b). The Col I hydrogel exhibited a steady increase, indicating a gradual deposition of proteoglycan-rich matrix. In contrast, the Col I/II-HA hydrogel showed unstable expression, peaking on day 21 before dropping. The disappearance of *ACAN* expression by day 28 suggests that the environment favored progression toward an osteogenic rather than a chondrogenic phenotype. On the other hand, the presence of chondrocytes in the Col I/II-HA+Cho resulted in a similar expression pattern to that observed in the Col I hydrogel, exhibiting a steady increase in *ACAN* expression from day 7 to day 28. The expression on day 28 was significantly higher compared to the Col I/II-HA hydrogel, suggesting that chondrocytes enhanced the chondrogenic microenvironment during the late stages of differentiation. Interestingly, the highest *ACAN* expression was observed in the Col I/II-HA+Cho_only hydrogel, indicating that the chondrocytes actively maintained their phenotype despite the osteoinductive medium and were the main contributors to *ACAN* production. However, this effect was diminished when chondrocytes were co-cultured with hMSCs and HUVECs in the Col I/II-HA+Cho hydrogel. This reduction could be due to the presence of hMSCs, which can downregulate chondrocyte differentiation. Xu et al.^68^ reported a similar observation, showing that co-culturing with hMSCs reduced the deposition of ECM components by rat articular chondrocytes.

The expression profiles of *MMP13* (Fig. 5a) and *VEGFA* (Fig. 5b) revealed more information about how hydrogel composition and cellular components influence chondrocyte hypertrophy and angiogenic signaling within the hydrogels. Both markers play crucial roles in the later stages of chondrogenesis, with *MMP13* involved in ECM remodeling and *VEGFA* promoting vascular invasion of hypertrophic cartilage. Their regulation reflects essential transitional processes in osteochondral development^69,70^. The *MMP13* expression pattern in the Col I hydrogel, which rises gradually until day 21 before declining, reflects active ECM remodeling associated with osteogenic and angiogenic development. Both MSCs and endothelial cells can secrete *MMP13*, which facilitates collagen turnover, endothelial invasion, and vascular sprouting during tissue formation^35^. A similar trend was observed in the Col I/II-HA hydrogel, albeit with significantly lower expression levels, suggesting that the inclusion of Col II and HA reduces hypertrophic signaling.

Interestingly, incorporating chondrocytes into the Col I/II-HA+Cho hydrogel significantly increased *MMP13* expression during the early culture phase (days 4 and 7) compared to the Col I/II-HA hydrogel. This suggests that direct interactions between hMSCs, HUVECs, and chondrocytes temporarily promote a more hypertrophic-like phenotype or accelerate early matrix turnover. Furthermore, the chondrocytes themselves may have contributed directly to *MMP13* expression, as suggested by a study by Yamamoto et al.^71^, which revealed that human chondrocytes isolated from healthy adults constitutively express and secrete *MMP13*. The subsequent decrease in *MMP13* expression was likely caused by transcriptional repression resulting from sustained *SOX9* activity, which suppresses hypertrophy-associated *MMP13* expression by inhibiting *RUNX2*-dependent transcription^72,73^. In contrast, the consistently low *MMP13* levels in the Col I/II-HA+Cho_only hydrogel suggest that the chondrocytes alone maintain a stable phenotype with limited hypertrophic activity under these conditions^71^.

Temporal fluctuations in *VEGFA* gene expression across all hydrogels are consistent with its known dual role as a factor associated with hypertrophy and angiogenesis^74,75^. *VEGFA* production by both MSCs and HUVECs is partially driven by hypoxia-inducible factor 1 alpha (HIF-1α) under low oxygen tension and by paracrine crosstalk between the two cell types^76^. In the Col I and Col I/II-HA hydrogels, the early- to mid-stage variations (days 4–21) may reflect alternating cycles of hypoxia-driven activation and feedback regulation of angiogenic signaling. Significantly higher *VEGFA* expression in the Col I/II-HA hydrogel on days 7 and 21 suggests that including Col II and HA enhances angiogenic signaling, potentially by supporting a more physiologically relevant microenvironment that promotes endothelial activation. This finding aligns with previous research showing that HA-rich matrices can increase *VEGFA* expression and secretion during tissue remodeling and neovascularization^77^. The addition of chondrocytes in the Col I/II-HA+Cho hydrogel resulted in significant *VEGFA* upregulation during the initial stages (days 4 and 7), suggesting that chondrocytes contribute to transient angiogenic or hypertrophic temporary signaling when cultured with hMSCs and HUVECs. This early increase may represent the initiation of matrix remodeling and vascular patterning rather than a sustained hypertrophic state, as expression levels subsequently stabilized. Conversely, the Col I/II-HA+Cho_only constructs, which contained only chondrocytes, exhibited consistently high *VEGFA* expression across all time points. Since the *VEGFA* gene transcription is driven by HIF-1α under low oxygen tension^76^, we hypothesize that its upregulation may result from hypoxic conditions within the hydrogel, arising from its relatively large diameter and thickness. Sasaki et al.^16^ demonstrated that cell aggregates with a diameter of 1.5 mm develop hypoxic cores. Since all of our hydrogels exceeded this size (Fig. 2), it is likely that similar oxygen gradients formed, although we did not directly measure them. Given the important role of hypoxia in EO, future studies will aim to incorporate and examine hypoxic conditions within our model.

Overall, these findings suggest that Col II and HA modulate chondrogenic, hypertrophic, and angiogenic signaling within the composite hydrogels. Their inclusion promoted early *SOX9* activation and transient chondrogenic differentiation, supporting the initial steps of EO. The addition of chondrocytes reinforced a chondrogenic microenvironment and temporarily enhanced hypertrophic and angiogenic signaling. This emphasizes their regulatory role in coordinating the cartilage-to-bone transition during osteochondral development. Together, these results emphasize the synergistic interactions between hMSCs, HUVECs, and chondrocytes, suggesting that the Col I/II-HA+Cho hydrogel can partially recapitulate key stages of native EO.

### ECM deposition and cell migration

Alcian blue staining was used to assess GAG distribution and cell migration in various hydrogels after 28 days of osteogenic differentiation (Fig. 9). In hydrogels containing hMSCs/HUVECs, that is, Col I, Col I/II-HA, and Col I/II-HA+Cho, cell migration from the bottom into the hydrogel was observed (Fig. 9, indicated by the orange arrows). Notably, greater cell migration occurred in the Col I/II-HA hydrogel, with a higher concentration of cells in the top layer compared to the Col I hydrogel (Fig. 9, indicated by pink arrows). This suggests that the Col I/II-HA hydrogel facilitated more extensive cell migration, likely due to the combined effects of Col II and HA, both of which promote cell movement and ECM remodeling^66,78^. In the Col I/II-HA+Cho hydrogel, cells were observed in the top section as well. However, it was unclear whether these cells had migrated from the bottom or if they were chondrocytes originally embedded in the hydrogel, now concentrated in the top and bottom layers.

Alcian blue staining further revealed differences in the intensity and distribution of the cartilaginous matrix across the hydrogels. Since HA is a type of GAG, it was also stained with Alcian blue^79^. Consequently, the hydrogels containing HA exhibited slightly darker (more intense) staining than the Col I hydrogel. In the Col I hydrogel, staining was most intense at the bottom, corresponding to the area where most cells were located. This suggests that the cells primarily remained in the bottom part of the hydrogel and were likely involved in limited ECM production. In contrast, both the Col I/II-HA and Col I/II-HA+Cho hydrogels showed more intense Alcian blue staining, not only at the bottom but also extending toward the top, indicating that these hydrogels promoted more extensive cartilaginous matrix deposition. The presence of chondrocytes in the Col I/II-HA+Cho hydrogel resulted in more intense Alcian blue staining that extended deeper into the hydrogel from the top and bottom (Fig. 9, indicated by the black lines), compared to the Col I/II-HA hydrogel. This indicates higher GAG production and the ability of the chondrocytes to secrete a cartilaginous matrix. However, the Col I/II-HA+Cho_only hydrogel, consisting only of chondrocytes, exhibited weak Alcian blue staining, suggesting that the absence of hMSCs may result in a less robust cartilaginous matrix. This highlights the importance of the interaction between hMSCs and chondrocytes in enhancing matrix deposition. Combining these two cell types likely creates a dynamic interaction that promotes more effective cartilage formation, indicating a potential synergistic effect. This finding is consistent with previous studies showing that co-culturing chondrocytes with other cell types, such as MSCs, enhances ECM production and tissue regeneration^80^.

### Mechanical properties of hydrogels

**Figure 10.**
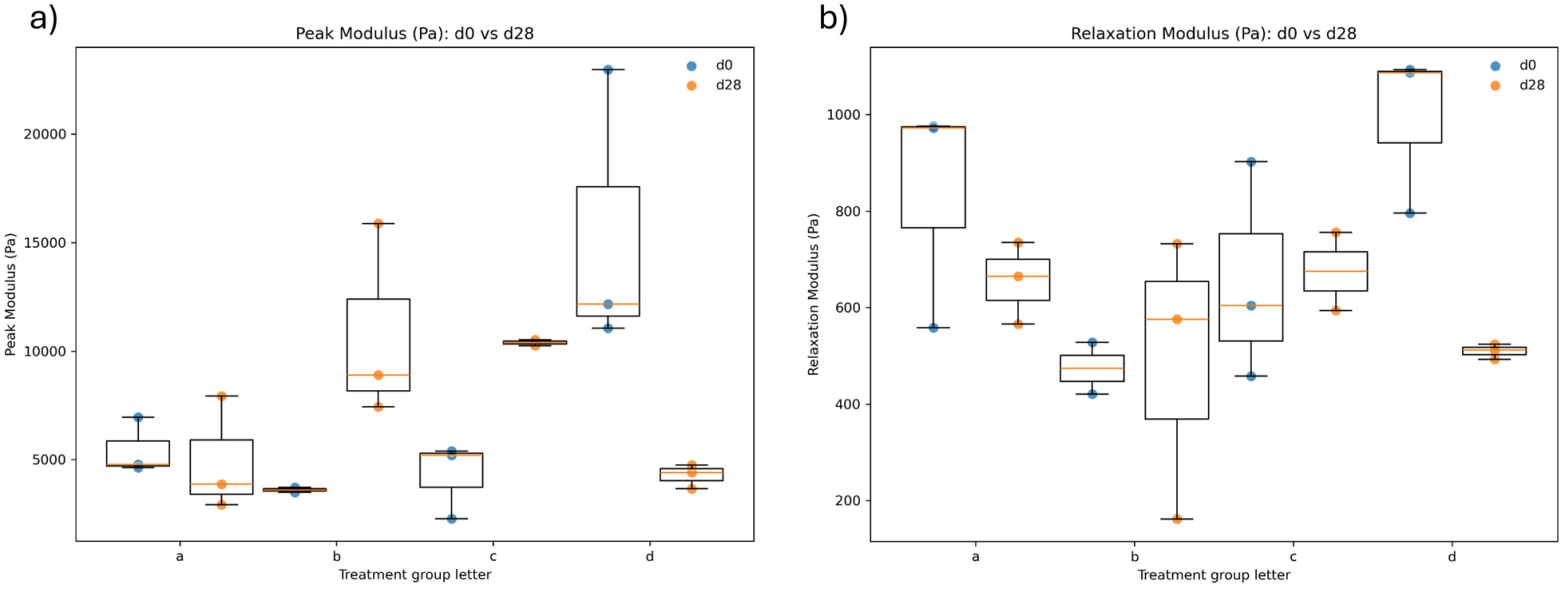
Hydrogel mechanical properties. (a) Peak modulus and (b) relaxation modulus of hydrogels marked with the following letters: a = Col I/II-HA, b = Col I/II-HA+Cho, c = Col I/II-HA+Cho_only, and d = Col I measured on day 0 (blue dots) and day 28 (orange dots) of osteogenic differentiation in the DIF medium. The data are presented in Pa as a box-and-whisker plot. The box represents the interquartile range, with the line indicating the median, and the whiskers show the minimum and maximum values.

The collagen hydrogels used have the mechanical properties within the range of Young’s modulus, from 0.5 to 27 kPa (Fig. 10)^81^. However, most of the studies rely solely on continuous compression test and measure the sample geometry with calipers. In our study, we used optical and force methods to measure the geometry of the samples. Nevertheless, our results fall within the observed range in the literature. The mechanical properties of the pure Col I hydrogel were superior to those of the Col I/II-HA hydrogel on day 0 of osteogenic differentiation. This implies that adding Col II and HA reduces the mechanical properties of the hydrogels. This aligns with a study done by Vázquez-Portalatín et al., who confirmed that adding Col II to Col I gels significantly decreases their stiffness. They attributed this decrease to a reduction in total protein concentration in the gel and an increase in void space^28^. However, the Col I hydrogel lost its mechanical properties over time, for peak and relaxation Young’s moduli. This suggests a lack of matrix to retain water and structural changes in the collagen matrix composition, probably caused by cell cultivation. According to Bacakova et al.^25^, cells cultured in Col I hydrogels exhibited contraction, shrinkage, and/or actuation due to the cells’ traction on the hydrogel surface. Additionally, cells cultured on hydrogels can alter the intrinsic mechanical properties of the hydrogels, such as compressive strain energy, tensile modulus, and compressive modulus^82^. The minimal loss of mechanical properties of the Col I/II-HA hydrogel after 28 days of differentiation in osteogenic medium may be due to the cells’ increased osteogenic and chondrogenic activity, which promotes ECM deposition.

Young’s modulus increased significantly from day 0 to day 28 for the Col I/II-HA+Cho and Col I/II-HA+Cho_only hydrogels containing chondrocytes in co-culture. This may be due to the chondrocytes depositing ECM that contributes to the hydrogel’s mechanical strength, which is in accordance with other studies^83^.

## Conclusion

This study highlights the potential to mimic the early stages of native EO through the co-culture of hMSCs, HUVECs, and human chondrocytes within Col I hydrogels enriched with Col II and HA. We demonstrated that incorporating Col II and HA into the hydrogels significantly improved osteogenic differentiation. Among the tested hydrogel formulations, the Col I/II-HA hydrogel promoted robust osteogenic responses. Late-stage markers also exhibited higher expression peaks in the Col I/II-HA hydrogel, indicating improved osteogenic maturation. The presence of chondrocytes modulated this process further, contributing to early peaks in late-stage osteogenic markers. Notably, chondrocytes cultured alone in the hydrogels expressed late-stage osteogenic markers, suggesting their active contribution to osteogenesis beyond merely supporting other cell types. Chondrocyte incorporation reinforced a chondrogenic microenvironment, promoting early *SOX9* activation and transient chondrogenic differentiation, which is consistent with initial EO events. Additionally, chondrocytes enhanced cartilaginous matrix deposition, as evidenced by intensified Alcian blue staining, and transiently upregulated hypertrophic (*MMP13*) and angiogenic *(VEGFA*) signaling, emphasizing their regulatory role in the cartilage-to-bone transition. However, reduced aggrecan gene expression in the presence of hMSCs suggests that, although co-culture enhances osteogenesis, it can also compromise the maintenance of a stable chondrogenic phenotype. These findings highlight the potential of fine-tuning matrix composition and cell-type interactions to guide lineage-specific differentiation, especially in early osteochondral development. Combining Col I and Col II, HA, and key cell types, such as hMSCs, endothelial cells (HUVECs), and chondrocytes, in hydrogels enhances osteogenic and chondrogenic differentiation, partially mimicking early stages of EO. Further research is needed to elucidate the underlying mechanisms, investigate marker expression at the protein level, and incorporate mechanical stimulation and hypoxic conditions to more accurately mimic the *in vivo* osteochondral environment. Nevertheless, our results demonstrate the potential of this approach for modeling the osteochondral interface and EO, as well as for advancing bone and cartilage regeneration strategies.
